# Spatial and Temporal Analyses of the Event of Death for 1480 in Milan Using the Data Contained in the Sforza’s Registers of the Dead

**DOI:** 10.3390/ijerph20042783

**Published:** 2023-02-04

**Authors:** Ester Luconi, Patrizia Boracchi, Riccardo Nodari, Francesco Comandatore, Giuseppe Marano, Folco Vaglienti, Massimo Galli, Elia Biganzoli

**Affiliations:** 1 Department of Biomedical and Clinical Sciences (DIBIC), University of Milan, 20157 Milan, Italy; 2 Data Science and Research Center (DSRC), University of Milan, 20157 Milan, Italy; 3 Romeo ed Enrica Invernizzi Paediatric Research Centre, Department of Biomedical and Clinical Sciences, University of Milan, 20157 Milan, Italy; 4 Department of Historical Studies, University of Milan, 20122 Milan, Italy

**Keywords:** Milano Sforza registers, historical epidemiology, spatial analysis, temporal analysis

## Abstract

Historical death registration was conducted primarily to assess the presence of plague. The *Liber Mortuorum of Milan* was one of Europe’s first registers with many socio-demographical details. In this work, we consider 1480 to make spatial and temporal analyses of the event of death to find possible explanations about the events’ distribution and the events’ trend over time. The spatial analyses involved Moran’s I, the LISA, and the heatmaps; the temporal analysis applied the Durbin-Watson test. All the analyses were conducted separately on all subjects (1813), children (765), and adults (1046). *Contrade* (districts) were considered for spatial analysis. Moran’s I and the Durbin Watson test were significant on all subjects and children’s analyses, and the LISA showed the same results for those groups. Children may significantly impact the distribution of death and the trend over time. At least half of the children were 0 years old, and survival in the very first childhood period was closely linked to the family, so that it could be a proxy of the conditions of an area.

## 1. Introduction

Books of deaths or *Necrologi* or *Liber Mortuorum* in different European cities were made to evaluate the possible presence of plague in the territory and to assess the spread of the disease among people. For example, from 1518, an epidemic period in London, all parish priests had to report the number of plague deaths in their parishes each week. Inspecting the body to determine the causes of death was the aim of the searchers, old women, who had to report to the parish clerk, who had the task of making counts that would be sent to the clerk of his company. Finally, the city-wide report based on the data was made for the Lord Mayor and aldermen and would be sent to the Privy Council the next day.

In 1592, another plague year, plague reports became public because large-scale broadsheet copies were posted in public areas [1].

The exceptionally high mortality of plague in the Mediterranean area in the fifteenth and sixteenth centuries led to the recording of the deaths in Barcelona. The recordings were made probably because the frequently recurrent epidemic was seen as a cause of his commercial decline, so taking an exact count of deaths from the plague would allow giving due weight to the phenomenon in the city. This goal has been achieved thanks to the letter carrier (*courreu)* that takes information about the number of deaths from city parishes [2].

In some Italian cases, the registration of the deaths also has other goals beyond monitoring the presence of plague; for example, in Florence, recordings were valuable in providing adequate provision for the city [3].

In addition, the *Liber Mortuorum* of Milan, which consists of 366 volumes presently held in *Archivio di Stato di Milano,* was made to monitor the possible presence of plague in the city. This historical source took place under Francesco Sforza in 1450 and was daily compiled until 1801, so it is probably the oldest established mortality register in a large European city, considering the socio-demographical information contained in it immediately from the first recordings, unlike other registers.

**Figure 1:**
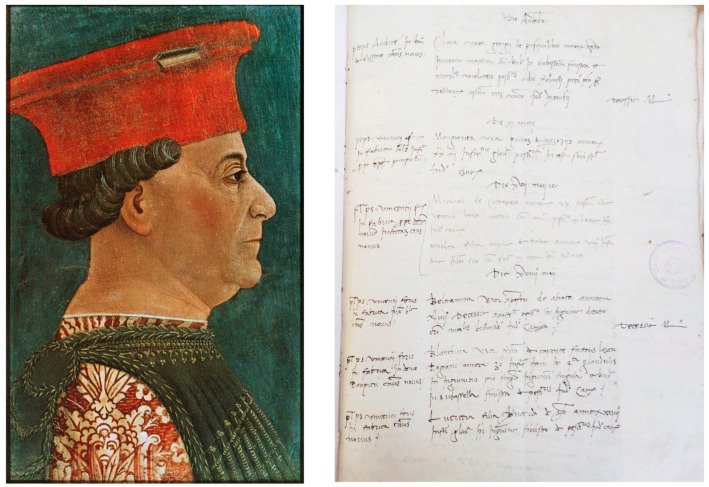
Francesco Sforza portrait by Bonifacio Brembo in 1460 (https://commons.wikimedia.org/wiki/File:Francesco_Sforza.jpg accessed on 20 December 2022) and a page of the the *Liber Mortuorum* of 1485 (courtesy from Luca Fois).

In Figure 1 is represented Francesco Sforza portrait by Bonifacio Brembo in 1460 and a page of the *Liber Mortuorum* of 1485.

During the fifteenth century, Milan had the most extensive urban population in Europe, estimated at 100,000 inhabitants [4]. In addition, it was a vital center of production and trade. It connected Europe and the rest of Italy [5].

The compilation of this historical source has involved a chain of people with different tasks, as will be described below.

The parish elders were the first link of the chain and were laypeople who represented a reference for the population of his community. They had various tasks: indicating to the civil authorities parishioners who needed help, monitoring environmental quality, supervising who produced polluting waste or sewage, and others. Regarding health control, they had the task of visiting the house [6] and taking socio-demographical information and the causes of death, the duration of the illness, and if the case was followed by a doctor or a surgeon (to have more information about the case). All that information was reported to the *Tribunale di Sanità.* The latter sent the *Catelano* (a doctor who specialized in detecting epidemic disease) to obtain a medical opinion about the cause of death [7].

The information contained in the fifteenth-century registries was: geographical information about the event (*Sestiere* and parish and the place where the event occurred if it is different from the home of the subject), the name, surname, or nickname, social and professional status of the deceased, age at death (expressed in years, months or days), the cause of death (rich in detail, for example, the duration of the disease or all the information about the incident in case of violent deaths), the name of who determined the cause of death.

After a plague period, it is recognized that there are changes at social, demographic, and economic levels due to the rapid and high decline in the population in a specific area. In particular, in periods immediately after the events, people and generations were less frail because of the selective effect of plague [8]. Thus, the study of the causes of death, the socio-demographical analysis, and the spatial analysis before and after a plague period can contribute to the study of the effect of the epidemy.

In addition, in a period with plague, the majority of the causes of death are probably caused by this illness. Only with the study of other periods is it possible to know the typical reasons for death of a historical period and the distribution of the event.

Spatial analysis can be used when there is a lack of information. For example, in chronic disease risk, the residential location can be used as a surrogate for unknown environmental exposures [9]. In a case where the socio-economic conditions in different parts of the city are mostly unknown, a typical situation of historical data, this analysis of the deaths could provide information about this context and, in addition, reveal spatial patterns.

Temporal analysis of the death event can be used to study if the socio-economic conditions of a particular area, or other conditions that have a connection with the event, improved over time or if the event has a relationship with seasonality.

This article aims to conduct geographical and temporal analyses of one year of the *Liber Mortuorum* of Milan, 1480, one of the rare periods characterized by peace and without epidemics; this period was considered representative of other periods with similar characteristics.

The spatial autocorrelation was conducted to assess if there is a spatial pattern of the density of deaths in Milan in 1480. The events’ density in different areas was assessed because of the lack of information about the precise number of people in Milan and the population distribution.

The temporal autocorrelation was conducted to assess if there is a temporal pattern of the number of death events.

The analyses were conducted on all subjects and children (under eight years old) and adults (at least eight years old). Child mortality is an essential indicator of the health status of an area. In modern times it can be used to compare health conditions between countries. In addition, perinatal and infant mortalities are associated with biological and socio-economic factors [10]. The analyses of adults were conducted to assess if the distribution of the event of death was mainly due to the death of children (the Renaissance period was probably marked by high infant mortality) or, in the case in which the mortality of adults followed the same pattern as the mortality of children, by a factor that affects both causes.

For the spatial analysis, it is crucial to consider the subdivision of Milan in *Contrade* (districts) because it will be based on them, and the number of deaths in each subdivision will be calculated regarding the number of events in parishes in each *Contrada*.

The territory of Milan was subdivided into an “*intus*” part (from roman walls to medieval walls) and a “*foris*” part (from medieval walls to Spanish walls). The *intus* part was subdivided into six *Sestieri*; each took a name from the corresponding door in the medieval walls. From north and clockwise, there were: *Nuova, Orientale, Romana, Ticinese, Vercellina,* and *Comasina.* The *Sestieri* had the form of irregular triangles, and the vertex was located near the *Palazzo della Regione*.

Each *Sestiere* was subdivided into five *Contrade*. The *Contrade* near the city center were called “noble”.

The *Sestieri* and the *Contrade* had a connotation of the military organization primarily: the troops in the *Sestieri* had a defensive purpose, at least in roman times, of the respective doors, and in the *Contrade* there were secondary watchtowers.

Each *Sestiere* had different traditions and cadences of the Milanese dialect [11], but the “real” fulcrum of the social organization was the parishes.

Each parish had its area of relevance; in some cases, it was also located outside the walls, so that a parish could have an *intus* area and a *foris* area.

## 2. Methods

Based on material found on the internet [12] a digitalization and a reconstruction of the distribution of the *Contrade* and the *Sestieri* in Milan was performed thanks to Qgis ver. 3.13.3 [13].

The position of the parishes contained in the registry of 1480 on a modern map has been identified thank to some material found online [14,15] because many of them no longer exist, the locations for those churches are approximative: for example, it was possible to have the name of the modern street in which they were but not the civic number. The number of the events in one *Contrada* was obtained as the sum of the deaths in the parishes in the *Contrada* and the number of the events in the *foris* area was obtained as the number of the events in churches outside the medieval walls. The density was obtained by (number of deaths /areas in square meters) * 1000 for both areas.

Moran’s I [16] allows us to evaluate the presence of the spatial autocorrelation of the density of deaths, so thanks to this index, it is possible, for example, to assess if there is a pattern of socio-demographic conditions that had an impact on the causes of death.

The spatial structure to calculate the global I was built up taking into consideration the spatially contiguous neighbors and the queen adjacency between the polygons of the *Contrade* [17]. In calculating the number of adjacencies between the polygon, the row standardization of the spatial weight matrix was contemplated.

The non-spatial matrix for calculating of the global I was made considering the territorial density.

Moran’s I is affected by outliers: the presence of the geographical ones was excluded because all polygons were very near each other. A square root transformation was performed in the case of outliers and asymmetry in the territorial density variable.

In the presence of autocorrelation expressed by Moran’s I, the spatial clusters’ locations were examined thanks to the local Moran’s I (LISA) [18].

Temporal autocorrelation is the relationship between successive values of the same variable, in this case, the number of deaths.

Temporal autocorrelation was assessed using the Durbin-Watson test [19]; the number of deaths for each week was considered as the response variable, and the time as the independent variable. The assumptions are: normally distributed errors with a mean of 0 and the stationarity of the errors. The first assumption was checked by a normal quantile quantile plot and the second by the Dickey-Fuller test. The subdivision of the time was the same for all subjects, children, and adults, to evaluate the presence of temporal autocorrelation in the same time intervals.

The relationship between the number of deaths each week and the time was not linear (based on graphical representations), so it was used a restricted cubic spline with four knots. For the analysis, it was considered that our data started on 23 January.

To analyze if the *Contrade* have a peak of the density of the event in the same periods, heatmaps were used, and the temporal intervals were in months.

All the analyses were conducted using R (ver. 4.0.4) [20]. The proximity analysis was conducted using the library “rgdal” [21], “GISTools” [22] and “mapview” [23]. Library “spdep” [24] was used to perform the Moran’s I.

For the spatial local autocorrelation were used the packages “maptools” [25], “classInt” [26] and “gstat” [27,28]. Heatmaps were performed using the package “gplots” [29], Durbin Watson test were performed using the package “lmtest” [30]. Splines were performed using the packages “rms” [31].

## 3. Results

In the 1480 registry, there were 1813 subjects, 911 females and 902 males.

Considering all the subjects, the median age was 17 years old (min 0, max 100 years, first quantile: 1, third quantile: 50). For two subjects, the age was unknown.

For adults, the median age was 40 years (min 8, max 100 years, first quantile: 24, third quantile: 60).

For children, the median age was 0 years (min 0, max 7, first quantile: 0, third quantile: 2).

In the registry, there were 765 children (subjects under eight years old).

**Figure 2:**
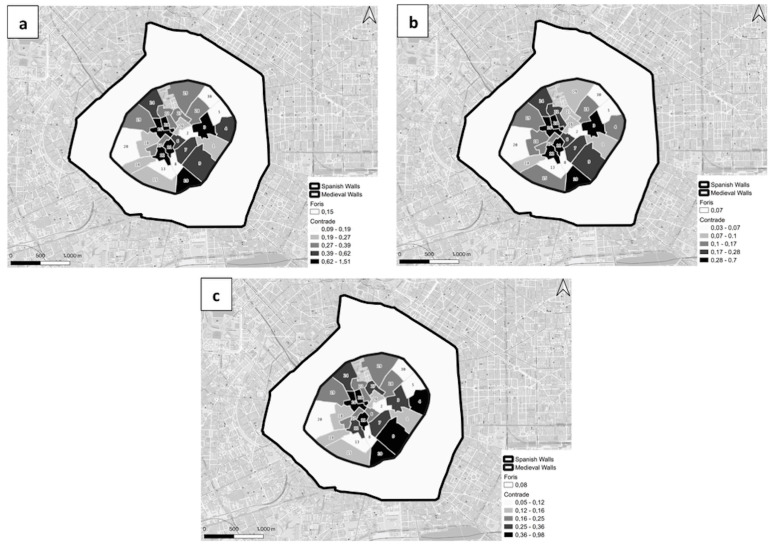
Distribution of the deceased people in the *Contrade* and the *foris* area for all subjects (**a**), for children (subjects under eight years old) (**b**), and adults (**c**). The names of the *Contrade* associated with the IDs are reported in Table 1.

Figure 2 represents the distribution of the deceased people in the *Contrade* and the *foris* area for all subjects (a), for children (subjects under eight years old) (b), and for adults (c).

It can be seen that in the *foris* zone there is a low distribution of the event; this can be due to the extension of the area in consideration. In addition, it was characterized by the *Cassine* (farmhouses) and craft activities. In 1412, this area was populated by modest workers, but there were also houses of prominent personalities [32]. On the other hand, the high distribution of the event in the *Contrade* 3, 21, 16, 11, and 12 could probably be attributed to the high density of the population. To our knowledge, Milan was characterized by social heterogeneity at the *Sestiere* and *Contrade* levels. However, it could be possible that the center of the city was a commercial area, also with homes of merchants and retailers; moving to the peripheral area, there was probably a residential area and a production area (textiles, weapons, and armor) near the medieval walls [33]. Table 1 presents, for each *Contrada* and the *foris* area, the surface expressed in square meters, the *Sestiere* to which it belongs, the number and the percentage of females, the number and the percentage of children (individuals under eight years old), and the median age with the interquartile range (IQR).

It could be noted from Figure 2 that the distribution of the event in children and all subjects is very similar. In particular, *Contrade* 3, 10, 11, 12, 16, and 21 are characterized by a high density of death of all subjects and children; it could be noted, from Table 1, that a relatively low age at death depicts the *Contrade* 3, 11, and 12. The high density of the event in other *Contrade* could be due to a possible high population density or a relatively small area.

The distribution of the event of death in adults is very similar to those for children and all subjects, but there is a high density in the *Contrade* near the medieval walls.

In Figure 2a–c it can be noted that there are some zones with a high density of event near zones with a high density of the event (for example *Contrade* 21 and 16 and *Contrade* 11 and 12. The latter does not show a high density in adults’ pattern) and zone with a low density of the event near zones with a low density of events (for example *Contrade* 5 and 30). Thus, there may be a spatial association between the events. Moran’s I statistic was used to assess it.

The global Moran’s I statistic for all subjects was 0.14, and the *p*-value was 0.039, so there is evidence against the null hypothesis (the presence of randomness of the territorial distribution of the death density). Furthermore, there is statistical evidence for the presence of spatial autocorrelation in a positive way (presence of a zone with a high value of the variable near other zones characterized by a high value of the variable and/or the presence of an area with a low value of the variable near different zones characterized by a low value of the density of deaths).

The Moran’s I test for children gave the following results: it was significant (*p* value = 0.032), and the value was 0.15, so there is a signal of the presence of spatial clusters.

The global Moran’s I test for adults is not significant (*p* value = 0.074), so there is no evidence of spatial autocorrelation.

**Figure 3:**
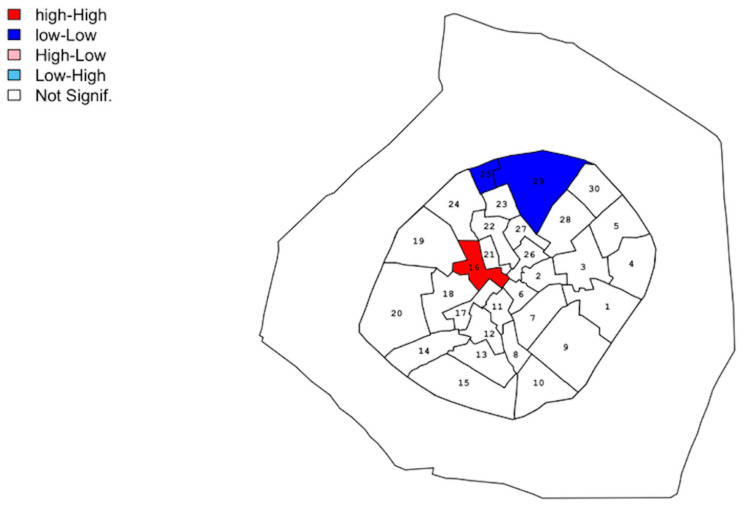
Position of clusters for all subjects and children in 1480. The distribution is the same for those two groups.

Moran’s I statistic is a global measure of spatial autocorrelation. Therefore, to evaluates clusters at local scale LISA (Local Index of Spatial Autocorrelation) was performed only where the global spatial autocorrelation was significant (so for all subjects, and adults). Figure 3 represents the clusters according to LISA for those two groups.

The *Contrade* marked by color are statistically significant on the LISA and represents a high correlation with the local Moran I of the neighboring *Contrade*.

There is a “hot spot” cluster (high-high) for all subjects in *Contrada* number 16, corresponding to Galleria Meravigli and Palazzo Turati zone. There is a “cold spot” (low-low) cluster in correspondence to *Contrade* 29 and 25 with a low value of the density of the events; it corresponds to the zone of Brera and Borgonuovo in Milan.

The clusters identified for children by the analysis were the same as the previous one.

Taking those results into account, it is possible that the pattern of mortality observed for all the subjects is mainly driven by children’s one.

**Figure 4:**
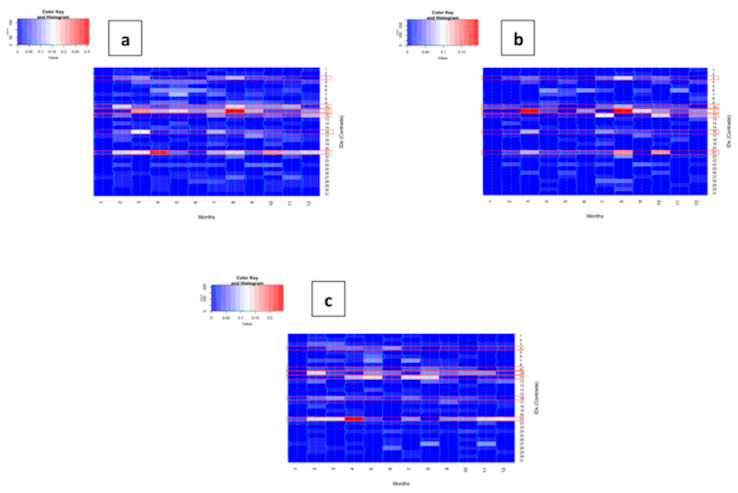
Heatmaps for all subjects (**a**), children (**b**), and adults (**c**). Horizontal red bands refer to the *Contrade* in which there were a high density of the events for the three groups of subjects.

Figure 4 represents the heatmap for all subjects (a), children (b), and adults (c). The heatmap was made to assess the number of deaths of each *Contrade* month by month.

It can be observed for all subjects that *Contrada* 21 has two peaks: one in April and one in October. Another *Contrada* has two peaks: one in March and one in August (*Contrada* 11). Other *Contrade* have a lot of less pronounced peaks.

Regarding children, *Contrada* number 21 had two peaks, one in August and one in October (so the peaks were different between children and all subjects); also, *Contrada* number 11 had two peaks: one in March and one in August, as for all subjects. Other *Contrade* had different peaks but with minor intensity.

For adults, it could be noted that there is only one *Contrada* with a great peak (number 21 in April) and other *Contrade* have a series of minor peaks.

The Durbin-Watson test for all subjects was significant (*p* = 0.003), so there is evidence against the null hypothesis. Furthermore, there is a presence of temporal autocorrelation. The value of this test is 1.30, so there is a positive autocorrelation.

The Durbin-Watson test for the temporal autocorrelation for children was significant (*p* value < 0.01), so there is evidence against the null hypothesis. Therefore, there is a presence of temporal autocorrelation. The value of the statistic was 0.93, so there is a positive autocorrelation.

The Durbin-Watson test for adults was not significant (*p* value= 0.204), so there was no evidence of temporal autocorrelation; the value of the statistic test was 1.78.

Considering those results, the temporal autocorrelation observed for all the subjects may be a consequence of the children’s one. Positive autocorrelation indicates that the number of deaths in a previous week has a “positive” impact on the considered week.

The trends of the number of deaths in weeks for all subjects, children, and adults are shown in Figure S1: it can be seen that the trends for all subjects and children are very similar.

## 4. Discussion

The definition of public health surveillance is “the ongoing, systematic collection, analysis, and interpretation of health-related data essential to planning, implementation, and evaluation of public health practice” [34]. In Milan, Duke Giangaleazzo Visconti (1351–1402) established a form of public health surveillance which made mandatory a notification system on all illnesses and deaths. Furthermore, he founded the figure of the health commissioner, which deals with compliance with health regulations and finally decrees the separation of the healthy from the sick, also thanks to spaces outside the city. This collection of information was useful later, in the plague of 1468, to assess that most of the cases were present in the *Cinque vie* (Five Points or Five Streets) area; some links in the chain of contagion in other parts of the city can be traced back to this area. The leading public health practice was the separation of the healthy from the sick [35].

We have chosen to perform spatial and temporal analyses of 1480, a period without war and epidemics, of the *Liber Mortuorum* of Milan to study the distribution of the death event in the city and during the year. This source is extraordinary because it is, to our knowledge, the most ancient European registry that contains socio-demographical data at the individual level.

There is no documentation about the distribution of the population in the considered time and the socio-economical differences in parts of the city.

However, the registration of the death also allows the socio-economic conditions of a certain area to be assessed: Edwin Chadwick (1800–1890) declared that poverty and disease are closely related, and Lemuel Shattuck (1793–1859) report that living conditions have an important impact on infant and maternal mortality and morbidity [36].

From the spatial and temporal analysis, it is clear that infant and child deaths rule the pattern of the first and the second trend.

In the 1480 registry, there is a scarcity of information about the causes of death in children.

As it was written in the introduction, children’s death is an important indicator to assess the health condition of an area. The very first period of childhood was a very precarious condition: lack of hygiene, contaminated water, malnutrition, and other conditions led, for example, to worms, diarrhea, and smallpox.

Noble children are generally destined for a future of marriage, religious life, or conduits; the destiny of non-noble children depended strictly on family’s finances, which was connected to the physical environment, the harvesting productivity, and the job market.

The nutrition of infants has a vital role in the growth and protection from illness: breast milk contains antibodies and many nutrients, but poor and malnourished mothers often do not have enough milk to feed the baby, and it is possible that the mother died in childbirth [37]. From the distribution of the age of children, it can be seen that at least half of them had 0 years old.

The fact that the spatial cluster for all subjects and the children were the same possibly reflects that in the *Contrade* there were poor conditions (or wealthy conditions for the low-low clusters), or it is possible that the areas were characterized by a high (low) density of population.

The territorial information linked to the causes of death can be very important to put an end to an epidemic phenomenon, such as what happened in a cholera epidemic in London during the nineteenth century, during which the study conducted by John Snow about the zones where the event took place allowed him to establish the causes of the phenomenon (contaminated water) and to put in place measures to stop it [38].

The presence of temporal autocorrelation can be because there are illnesses affected by seasonality. For example, based on an analysis of the causes of mortality (currently in progress), many cases of respiratory diseases are in spring and winter, and diarrheal disorders are in summer and autumn. It is also possible that there are unknown conditions that have a connection with the event of deaths increasing over time.

Regarding the heatmaps, there is a major peak of deaths in *Contrada* 21 for April for all subjects and adults, so it is possible that this peak is ruled by the mortality of the adults, but in October there is a peak for children and all subjects, so it is possible that in this month there was a particular condition that affected children in this area. *Contrada* 11 had a high peak in August for all subjects and children.

Temporal analysis of the death event is essential in public health surveillance: comparing all causes of mortality in the same periods for different years allows excess mortality, which can inform public health actions; the UK Health Security Agency put in place weekly all-cause mortality surveillance [39].

More generally, the study of one year can help to understand if there are peaks due to the seasonality on the causes of death: a historical registry to study the pattern was the Florentine Dowry Found, established on February 1425 and five periods (1436–1439, 1448–1451, 1456–1459, 1477–1480, 1526–1531) were taken in consideration. They showed relatively low mortality during the winter and high mortality in the summer. Although epidemics mainly occur in summer and autumn, in non-epidemic periods, the phenomenon is higher in summer than in winter. Autumn mortality level is similar to spring in the absence of major epidemics; otherwise, the first is greater than the second [40].

The study of death events in a period without epidemics can have great importance in assessing the impact of the plague on the city. For example, it is possible to analyze the excess of mortality on age by comparing the distribution of the age of death, sex, and socioeconomic in plague or non-plague periods. Studies are generally in accordance that there is not a relevant difference in the sex distribution of plague victims, only some differences were found for the fifteenth century in Milan, but it is possible that it was due to the poor condition of migrant women and widows. The dying concerning socioeconomic factors changed over time: during the Black Death, plague was recognized as a universal killer; during the fifteenth century, poor people were mainly involved; and finally, during the seventeenth century, the rich people [41].

## 5. Conclusions

The Sforza Registers of the Dead is one of the first European registers containing detailed information at the individual level. In this work we considered the data of 1480, a year without plague and characterized by peace, to make spatial and temporal analyses of the death event. Spatial analyses of this event can reveal patterns with a connection to the socio-economic conditions in different parts of the city. Temporal analysis can reveal changes in the socio-economic conditions in time or a seasonal pattern.

Analyses reveal that children’s deaths could have an important impact on the spatial pattern and temporal trend of the events; this is interesting because children’s mortality is an indicator used to assess the health condition of a territory.

Considering a representative period of the event death in periods without plague can lead to some considerations on the impact of the plague in the same population in a period close to the one studied; for example, if the plague causes the age distribution to shift or if it mostly affects the districts where there has been a high density of death event from all causes in a period without epidemics (which are therefore districts with conditions of poverty and/or densely populated) or leads to a different temporal trend of event.

The use of all-cause mortality in spatial analysis and the trend over time can help to analyze the health status of the population in different districts and the entire city in modern time. An example is given by an analysis of Genoa [42].

The registration of the death, its cause, the subject’s socio-economical characteristics, and the territorial information are important to evaluate the health control situation and to do the contact tracing in case of epidemic diseases (plague of 1468 and the *Cinque vie*). Registers are also valuable in finding epidemics in the history of Milan, and they can be used to study the interaction between politics and sciences in different periods [43,44]. The registers, in this sense, are the first examples of a monitoring system that is also used in modern times (COVID-19).

## Figures and Tables

**Table 1:** The distribution of gender and age in each *Contrada* and the *foris* area.

ID	Name	Sestiere	Surface (m^2^)	Number of Females (%)	Number of Children (%)	Age Median (IQR)
1	Contrada del Verzaro	Orientale	126,794	16 (57.1%)	11 (39.3%)	27.0 (56.50)
2	Nobile Contrada delle Farine	Orientale	39,551	4 (80%)	2 (40.0%)	36.0 (29.00)
3	Contrada dell’Agnello	Orientale	101,678	31 (48.4%)	34 (53.1%)	5.0 (51.50)
4	Contrada della Cerva	Orientale	110,032	31 (52.5%)	19 (32.2%)	24.0 (47.00)
5	Contrada di Bagutta	Orientale	91,946	8 (53.3%)	4 (26.7%)	40.0 (62.00)
6	Contrada del Falcone	Romana	32,892	11 (73.3%)	9 (60.0%)	1.0 (24.00)
7	Nobile Contrada della Cicogna	Romana	89,414	21 (43.8%)	17 (35.4%)	12.0 (42.50)
8	Contrada del Fieno	Romana	35,705	2 (50.0%)	1 (25.0%)	29.5 (50.75)
9	Contrada del Brolo	Romana	195,466	51 (43.6%)	39 (33.3%)	25.0 (54.25)
10	Contrada delle Capre	Romana	104,510	53 (52.0%)	37 (36.3%)	22.0 (48.00)
11	Contrada della Lupa	Ticinese	35,742	28 (59.6%)	25 (53.2%)	5.0 (28.50)
12	Nobile Contrada di Sant’Ambrogio	Ticinese	49,210	11 (33.3%)	17 (51.5%)	6.0 (36.00)
13	Contrada delle Cornacchie	Ticinese	74,943	5 (62.5%)	4 (50.0%)	6.0 (34.50)
14	Contrada del Torchio	Ticinese	86,363	11 (57.9%)	8 (42.1%)	16.0 (43.50)
15	Contrada della Vetra	Ticinese	184,884	19 (38.8%)	21 (42.9%)	22.0 (49.00)
16	Contrada della Piscina	Vercellina	55,191	21 (55.3%)	16 (42.1%)	16.5 (52.75)
17	Nobile Contrada della Rosa	Vercellina	39,776	6 (40.0%)	5 (33.3%)	20.0 (31.50)
18	Contrada dei Morigi	Vercellina	96,595	11 (50.0%)	10 (45.5%)	18.5 (44.75)
19	Contrada della Porta	Vercellina	166,107	26 (52.0%)	20 (40.0%)	21.0 (59.00)
20	Contrada del Nirone	Vercellina	233,818	10 (45.5%)	10 (45.5%)	24.0 (59.50)
21	Nobile Contrada del Cordusio	Comasina	28,490	26 (60.5%)	15 (34.9%)	15.0 (54.00)
22	Contrada del Rovello	Comasina	57,225	10 (52.6%)	10 (52.6%)	4.0 (45.00)
23	Contrada dell’Orso	Comasina	44,126			
24	Contrada del Campo	Comasina	110,958	30 (56.6%)	25 (47.2%)	10.0 (49.00)
25	Contrada dei Fiori	Comasina	28,019			
26	Contrada Capitana	Nuova	36,023	6 (75.0%)	3 (37.5%)	12.5 (24.00)
27	Nobile Contrada dei Bossi	Nuova	39,388	7 (50.0%)	4 (28.6%)	36.5 (67.50)
28	Contrada della Mazza	Nuova	99,909	16 (44.4%)	15 (41.7%)	13.0 (59.00)
29	Contrada degli Andegari	Nuova	196,947	26 (49.1%)	19 (35.8%)	20.0 (49.00)
30	Contrada della Spiga	Nuova	75,919	3 (37.5%)	3 (37.5%)	18.5 (43.25)
	FORIS		5,452,942	411 (50.2%)	362 (44.2%)	13.5 (48.00)

## Data Availability

The data are not currently publicly available because the development of the database is still ongoing. However, we will communicate the data repository link when it will be ready.

## Supplementary Materials

The following supporting information can be downloaded at https://www.mdpi.com/article/10.3390/ijerph20042783/s1, Figure S1: Trend of the death event for children, adults and all subjects.
