# Supplementary figures and images for: Spatial and Temporal Analyses of the Event of Death for 1480 in Milan Using the Data Contained in the Sforza’s Registers of the Dead
Source: Int J Environ Res Public Health. 2023 Feb 4;20(4):2783. doi: 10.3390/ijerph20042783 (PMC9956338; doi:10.3390/ijerph20042783)

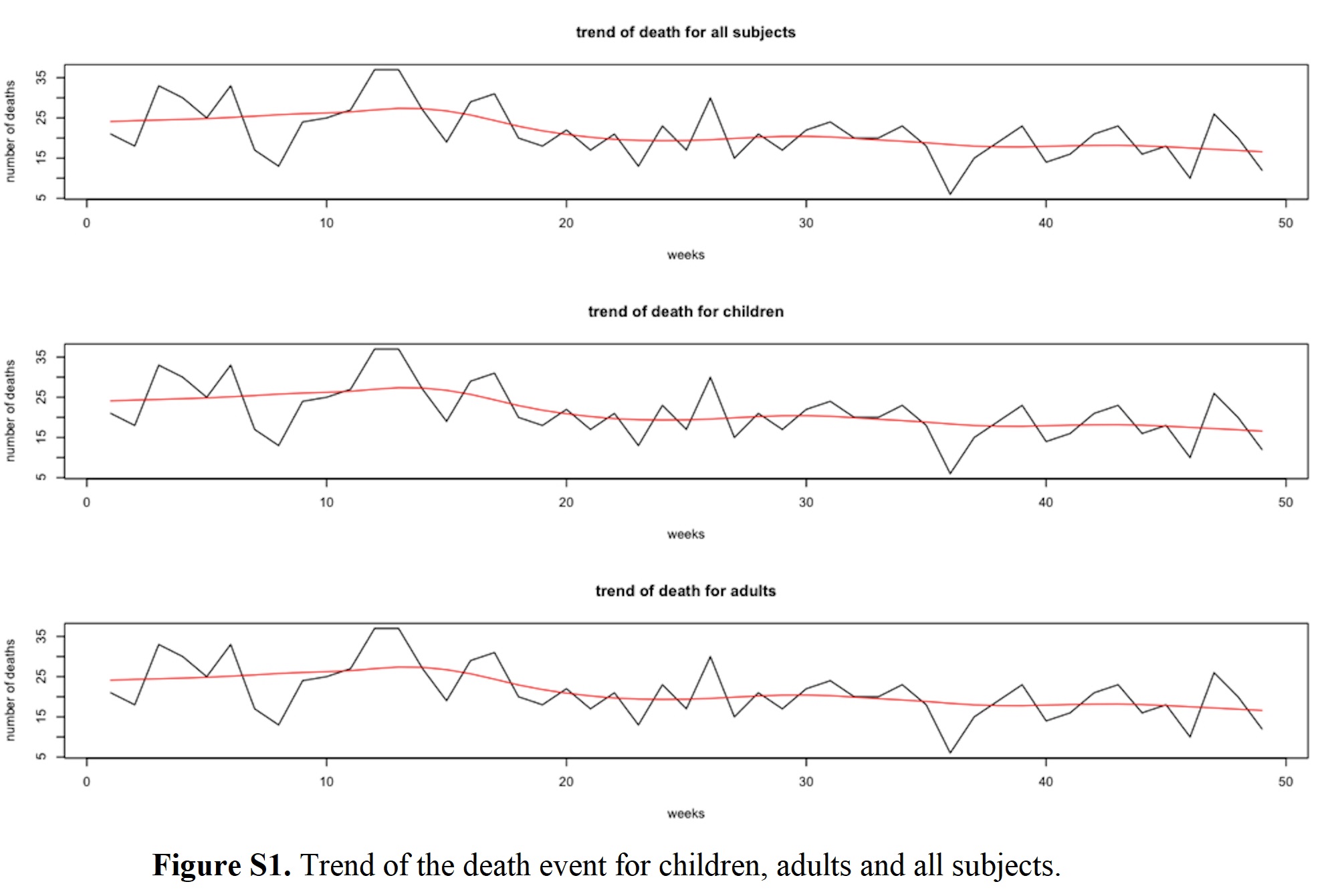

Supplement: Supplementary file 1 [file ijerph-20-02783-s001.zip › ijerph-2144821-supplementary Figure S1.jpg]
